# Reliable estimation of antimicrobial use and its evolution between 2010 and 2013 in French swine farms

**DOI:** 10.1186/s40813-018-0084-7

**Published:** 2018-04-17

**Authors:** Anne Hémonic, Claire Chauvin, Didier Delzescaux, Fabien Verliat, Isabelle Corrégé, The French Working Group ‘antimicrobials in the swine industry

**Affiliations:** 1 IFIP-Institut du porc, Domaine de la Motte au Vicomte, 35104, 35651 Le Rheu, BP France; 2 Anses Laboratoire de Ploufragan-Plouzané, 53, 22440 Ploufragan, BP France; 3 Inaporc, 149 rue de Bercy, 75595, 12 Paris Cedex, France

**Keywords:** Swine, Antimicrobial consumption, DDD, ALEA, ‘One health’

## Abstract

**Background:**

There has been a strong implication of both the French swine industry and the national authorities on reducing the use of antimicrobials in swine production since 2010. The annual monitoring of antimicrobial sales by the French Veterinary Medicines Agency (Anses-ANMV) provides estimates but not detailed figures on actual on-farm usage of antimicrobials in swine production.

**Results:**

In order to provide detailed information on the 2010 and 2013 antimicrobial use in the French swine industry, the methodology of cross-sectional retrospective study on a representative sample of at least 150 farms has been elected. The analysis of the collected data shows a strong and significant decrease in antimicrobial exposure of pigs between 2010 and 2013. Over three years, the average number of days of treatment significantly decreased by 29% in suckling piglets and by 19% in weaned piglets. In fattening pigs, the drop (− 29%) was not statistically significant. Only usage in sows did increase over that period (+ 17%, non-significant), which might be associated with the transition to group-housing of pregnant sows that took place at the time. Also, over that period, the use of third- and fourth generation cephalosporins in suckling piglets decreased by 89%, and by 82% in sows, which confirms that the voluntary moratorium on these classes of antimicrobials decided at the end of 2010 has been effectively implemented.

**Conclusions:**

The methodology of random sampling of farms appears as a precise and robust tool to monitor antimicrobial use within a production animal species, able to fulfil industry and national authorities’ objectives and requirements to assess the outcome of concerted efforts on antimicrobial use reduction. It demonstrates that the use of antimicrobials decreased in the French swine industry between 2010 and 2013, including the classes considered as critical for human medicine.

## Background

Antimicrobial resistance is a global concern that ought to be tackled by a ‘One Health’ approach [1]. In the veterinary domain, this has driven the implementation of monitoring programmes of antimicrobial resistance levels and antimicrobial usage [2], especially in Europe [3, 4], where sales data are collected in nearly all Member States [3].

In France, sales data on antimicrobial veterinary medicinal products (VMPs) have been collected yearly since 1999 by the National Veterinary Medicines Agency (Anses-ANMV). Data were first expressed as volumes (tons of active compounds), before being converted in exposure units [4, 5]. Since 2009, attribution of VMPs sales to each animal production species is provided by manufacturers [5]. In 2011, the French pig farmers’ representative bodies, together with the French swine veterinary practitioners’ associations, voluntarily implemented a consensus decision to limit prescription and usage of all third- and fourth generation cephalosporins.^1^ This voluntary decision reserved the prescription of such antimicrobials to emergency cases, where the health of the animals was otherwise compromised and no alternate solution was at hand. In the meantime, the French Interprofessional Pork Council (Inaporc) wished to measure the compliance level of this voluntary moratorium, to measure the actual quantities of antibiotics used in the French pig sector (to compare them with species-specific estimates from the official yearly consumption figures from Anses -ANMV) and to gain enough detailed information to analyze the treatment patterns by animal category and indication for treatment. These objectives had to be fulfilled by adopting a pragmatic approach (i.e. without engaging into an exhaustive continuous prescription recording system).

With the scientific support of both the French Food Safety Agency (Anses) and the French Institute for pig and pork industry (Ifip), the working group opted for implementing a cross-sectional retrospective survey method within a representative sample of farms, which would allow rigorous data collection and provide reliable and detailed consumption estimates.

The objectives of this study were to describe the implementation of these surveys, to detail the consumption data and analyse the evolution of antimicrobial usage over a three-year period (2010–2013).

## Methods

The European Medicines Agency, within the framework of the European Surveillance of Veterinary Antimicrobial Consumption (ESVAC) project, recommended recently two methodologies for collecting data by animal species [6]: the implementation of a full coverage system (“census model”) or of studies in a representative sample of farms. The latter method allows to estimate national consumption by species, weight-group and production category. Therefore, this was the approach chosen in 2011 and 2014 by the French working group ‘antimicrobials in the swine industry’, through the selection of farm samples representative of the national production in 2010 and 2013, respectively.

### Farm random sampling

Previous on-farm surveys had been performed in Brittany, where over 50% of the national swine production is concentrated [7]. From these data,^2^ it was calculated that 127 randomly selected farms would provide a 15% precision level. In order for the samples to be representative of the French swine production, simple random sampling was then performed in the exhaustive national swine database of identification, BDPORC,^3^ of which were excluded boar studs, farms located in Corsica and in the French overseas territories. Of those were selected farms with more than 49 sows, and farms with less than 50 sows but with more than 99 places in postweaning and/or fattening units, to exclude backyard pigs owners from the sampling frame. The working group deliberately elected to maximise the size of the sample, and an objective of 150 farms was defined for each survey.

The representativeness of these samples was checked post hoc (Chi^2^) through confrontation of the farms characteristics (self reported production orientation, location in Brittany – the densest swine production area in France, membership to a production structure and number of sows) to those in the whole BDPORC database and to the national agricultural census.

### Farm inclusion process

In order to reach the pre-established target of 150 farms per studied year in 2010 and in 2013, 270 and 300 randomised farms were extracted from the national database, respectively.

For each sampled farm, an explanatory letter detailing the study’s principles and objectives was mailed to the corresponding production structure and treating veterinarian (as recorded in the database). Then, these professionals presented the information to the farmers, who decided – or not – to participate. Participation was voluntary and was not object to any form of compensation.

### Farm data collection

The process of data collection was segmented in three steps.

Upon reception of the farmers’ acceptance, a questionnaire was mailed to them and/or a phone survey was performed, in order to collect the data needed for the calculation of the antimicrobials used on their farm the previous year. The collected data were:

- The technical and/or economical records^4^ allowing to estimate the farm’s animal population potentially exposed to antimicrobials: number of sows, number of sold/slaughtered piglets/pigs.
- The complete list of rights-holders that had dispensed veterinary drugs and/or medicated feed to the farm that year.

The latter designated structures were then contacted, and asked to send the detailed list of VMPs containing antimicrobials that had been sold to each voluntary farmer that year. The lists were required to mention the complete products’ name, presentation including concentration, and quantities dispensed. For medicated feed, volume (tons), active substance(s) included in the feed and proportion (in ppm) were asked for.

Finally, detailed data on the way farmers used VMPs were collected through phone interviews. Questions targeted:

- The treated production stage(s): sows (including boars/gilts), suckling piglets, weaned piglets or fattening pigs. When the same VMP was administered to several production stages, the number of commercial units was dispatched according to the farmer’s declaration.
- The indication for the usage: the farmers were asked to mention the cause(s) of each treatment administered with the VMPs recorded (up to three indications per notified treatment were accepted). Each declared cause was later reclassified into one of the nine following indications: digestive, respiratory, locomotion, nervous, genito-urinary, cutaneous, udder/lactation disorders, or systemic condition. All types of treatments (i.e. individual as well as group treatments) were taken into account.

### Indicators for antimicrobial consumption

The amount of active substance used on a given farm was derived from the quantity of each VMP declared as dispensed to the farmer. Antimicrobial usage on that farm was secondarily expressed in different indicators, since no antimicrobial exposure unit was harmonised in Europe at the time [5, 8]:

- The number of course dose per animal, either produced (piglets, weaners, fatteners) or present (sows) (nCD/ animal), was calculated by the equation:

1. {[(quantities of active substance in mg)/(dose in mg/kg/d x duration in d x weight group in kg)]/number of animals}, where d is for days;

- The number of daily dose per produced or present animal (nDD/ animal) was calculated by the equation:

[2] {[(quantities of active substance in mg)/(dose in mg/kg/d x weight group in kg)]/number of animals}.

For these calculations, the dosage and duration values used were those set by the French veterinary medicines agency [9]. They have been defined for each VMP and take in consideration the highest dosage and duration values that are mentioned for swine in each VMP’s summary of product’s characteristics (SPC). The weight groups selected were 250 kg for a sow, 2 kg for a suckling piglet, 15 kg for a weaner and 50 kg for a fattener (live-weight).

The third selected indicator was the ALEA (Animal Level of Exposure to Antimicrobials), which is used by the national authorities to report on the yearly monitoring of antibiotic sales [8]. It is calculated as follows:

[3] {[(quantities of active substance in mg)/(dose in mg/kg/d x duration in d)]/biomass in kg}, where biomass is the sum of the number of sows times 300 kg, plus the number of slaughtered finishers times 105 kg, plus the number of culled sows times 350 kg.

The ALEA value was calculated for each sample of farms.

In addition, the frequencies of farms dispensed with given antimicrobial classes at given production stages were also retrieved from the collected data.

This multi-indicator approach allows to calculate usage levels at farm as well as at sample levels, for each production stage, for each administration route, for each antimicrobial class and indication of treatment.

### Data analysis

The disparity in antimicrobial usage between farms was explored with the Lorenz curve and captured through the proportion of high-consuming farms (proportion of farms that consume more than 50% of total nCD/animal in a given year per production stage).

The collected data were compared for each studied year (2010 and 2013) to those provided by the national monitoring programme [9, 10] overall and for each antimicrobial class, using the ALEA.

To detail the evolution of antimicrobial quantities used per production stage, antimicrobial class and indication, the number of daily doses per animal of both farms samples (2010 and 2013) were compared. Also, the proportions (frequencies) of farms using each antimicrobial class, or each treatment route, or concerned by each indication were compared with a Chi^2^ test. Quantitative results expressed with non-normally distributed indicators (e.g. nDD/animal) were compared through a Kruskall-Wallis test.

A 5% threshold was selected for the designation of a statistically significant difference. The SAS software was used.

## Results

### Constitution of the samples of farms

Both samples were within the predetermined target of 150 farms per studied year: 171 farms in 2010 and 157 in 2013 accepted to participate and provided near-complete data. In both cases, the participation rate was over 75%: 45 and 53 farmers declined to be included in the samples, respectively (other farms were excluded from the samples because they did not meet inclusion criteria: on-going depopulation or works on site and change in the production type with variation of the numbers of produced animals). Furthermore, nine of the farmers included in the 2013 sample declined to answer to the part of the phone survey detailing the causes of treatments (due to time constraints).

The representativeness of both samples, when assessed against the data of their respective national agricultural census, was confirmed (Table 1).

**Table 1:** Characteristics of the farms samples studied in 2010 and 2013, and comparison to the national data

		2010		2013	
		national reference	farm sample	national reference	farm sample
Farms located in Brittany^a^		51%	46%	49%	45%
Member of a production organisation^a^		83%	84%	85%	89%
Production orientation	Farrowing or farrow-to-weaning	7%	6%	5%	5%
	Farrow-to-finish	46%	47%	45%	45%
	Post-weaners, post-weaners to finish or finishers	47%	47%	50%	50%
Number of sows	5 to 49	Not available		9%	9%
	50 to 99			10%	10%
	100 to 199			42%	42%
	200 and more			39%	39%

^a^National reference data from the national database BDPORC, extracted for years 2010 and 2013. Other national reference data are from the department of statistics of the French Ministry of Agriculture. There was no statistical significant difference, within each year, between the sample’s criteria and those of the national reference (Chi-2 test)

### Overall estimation of the swine exposure to antimicrobials

The 2010 national survey of antimicrobial sales reported an ALEA of 1.22 [9] whereas the farm sample provided an ALEA of 0.88 for that same year. This difference is essentially explained by four antimicrobial classes: in the sample, antimicrobial exposure to tetracyclines, macrolides, sulphonamides and trimethoprim is respectively 36%, 37%, 48% and 47% lower than in the pre-mentioned national sales monitoring report.

The 2013 farm sample provides an ALEA of 0.82. For that same year, the national report on antimicrobial sales calculates an ALEA of 0.96 [5]. Most of this difference is explained by one antimicrobial family: in the sample, exposure to tetracyclines is found 26% lower than in the national sales monitoring report.

The difference between the exposure values obtained by the two methods was not statistically significant (*p* > 0.05) and tended to decrease (28% higher in the 2010 national monitoring data, and 16% higher in 2013).

The comparison of exposure levels three years apart shows a significant reduction in antimicrobial usage (*p* < 0.001) either through the national monitoring programme (− 21%) or the sample surveys (− 7%).

### Relative importance of the production stages in antimicrobial usage

Sow treatments weighed 1% of the total treatments recorded (nCD/animal) in both samples (i.e. 2010 and 2013). Suckling piglets accounted for 30% of the amount of nCD/animal in 2010 and 24% 3 years later. Weaned piglets accounted for 59% (in 2010) and 64% (in 2013) of the amount of nCD/animal, making the post-weaning stage the object of the majority of treatment. Finishers represented 9 and 11% of the amount of nCD/animal in 2010 and 2013, respectively.

### Evolution of usage in sows

Antimicrobial usage in sows was found to increase between 2010 and 2013 (+ 17% of nDD/animal, *p* > 0.05). There was however a statistically significant decrease in the usage of cephalosporins over the same period (− 80%, *p* < 0.05) (Table 2). There was a reduction of the proportion of high-consuming sow-farms (from 20% to 13%), which did not reach statistical significance.

**Table 2:** Use of antimicrobials in 2013 per production stage, reason for treatment; comparison with the 2010 data

	Antibiotic usage in sows				Antibiotic usage in suckling piglets				Antibiotic usage in weaned piglets				Antibiotic usage in fattening pigs			
	% nDD/an., 2013	Evolution of nDD/an. 2010-2013	% of using farms in 2010 and 2013		% nDD/an., 2013	Evolution of nDD/an., 2010-2013	% of using farms in 2010 and 2013		% nDD/an., 2013	Evolution of nDD/an., 2010-2013	% of using farms in 2010 and 2013		% nDD/an., 2013	Evolution of nDD/an., 2010-2013	% of using farms in 2010 and 2013	
			2010	2013			2010	2013			2010	2013			2010	2013
Sample size^a^ (farm number) in 2010 and 2013	2010 = 91 - 2013 = 79				2010 = 91 - 2013 = 79				2010 = 122 - 2013 = 116				2010 = 160 - 2013 = 146			
Classes of antimicrobials																
Aminosides	2%	0%	36%	38%	7%	-39%	27%	29%	6%	-23%	44%	47%	4%	40%	25%	21%
Cephalosporins (3-4Gs)	0%	-80%	11%	1%	1%	-89%	18%	4%	0%		2%	2%	0%		4%	2%
Fluoroquinolones	4%	-34%	53%	57%	8%	-36%	44%	41%	0%	60%	32%	23%	1%	-25%	24%	19%
Lincosamides	0%	-25%	8%	9%	3%	-63%	5%	4%	3%	-51%	32%	21%	10%	264%	17%	13%
Macrolides	8%	9%	57%	56%	3%	-37%	8%	13%	12%	-19%	49%	35%	19%	-54%	41%	37%
Polymyxins	1%	-80%	35%	30%	33%	-11%	42%	42%	39%	-22%	89%	80%	6%	-77%	21%	15%
Penicillins	8%	-27%	81%	82%	38%	-21%	64%	65%	6%	-8%	66%	60%	5%	-14%	61%	49%
TMP-sulphonamides	22%	39%	20%	18%	2%	-72%	4%	4%	22%	59%	27%	24%	15%	-30%	14%	12%
Tetracyclines	52%	62%	34%	44%	2%	40%	2%	4%	12%	-32%	52%	41%	38%	6%	44%	38%
Sample size^b^	2010 = 91 - 2013 = 79				2010 = 91 - 2013 = 79				2010 = 122 - 2013 = 116				2010 = 160 - 2013 = 146			
Pharmaceutical presentations																
Injectable forms	14%	2%	93%	92%	64%	-29%	82%	78%	1%	-4%	68%	67%	4%	8%	69%	62%
Oral powders, liquids and pastes^b^	61%	95%	40%	39%	17%	473%	14%	24%	26%	8%	64%	66%	60%	54%	43%	45%
Medicated feed premixes	24%	-36%	24%	18%	19%	-59%	14%	6%	74%	-25%	84%	73%	36%	-64%	29%	16%
Sample size^a^	2010 = 90 - 2013 = 74				2010 = 90 - 2013 = 79				2010 = 121 - 2013 = 107				2010 = 157 - 2013 = 141			
Indications for treatment																
Digestive	7%	29%	30%	34%	56%	-22%	67%	64%	62%	-29%	89%	88%	30%	-45%	36%	35%
Locomotor	3%	150%	29%	64%	37%	-17%	56%	58%	1%	-78%	28%	51%	1%	33%	35%	45%
Respiratory	11%	79%	22%	28%	5%	157%	6%	9%	19%	-6%	47%	50%	60%	-7%	63%	56%
Systemic	12%	69%	50%	58%	1%	-96%	14%	14%	12%	8%	36%	44%	7%	-32%	19%	24%
Genito-urinary	65%	17%	70%	70%	NA^c^	NA	NA	NA	NA	NA	NA	NA	NA	NA	NA	NA
Proportion of farms representing 50% of total usage	2010 = 20% - 2013 = 13%				2010 = 19% - 2013 = 14%				2010 = 25% - 2013 = 18%				2010 = 12% - 2013 = 8%			

^a^Sample size varies between weigh group, depending of the activity of the farms

^b^Only suckling piglets did receive oral paste formulations

^c^*NA* Not applicable

### Evolution of usage in suckling piglets

Parenteral antimicrobial usage in suckling piglets was significantly reduced (− 29% of nDD/animal) between 2010 and 2013 (*p* = 0.05) (Table 2). The most drastic decrease was observed for cephalosporins (− 89%). This reduction reflects the scarcity of this usage: 4% of the farms used cephalosporins in 2013, versus 18% in 2010.

### Evolution of usage in weaned piglets

Antimicrobial usage in weaned pigs decreased between 2010 and 2013 (− 19% of nDD/animal), a highly significant result (*p* = 0.006). The usage of medicated feed (directly reflected by premix) was reduced by 25%, whereas there was no significant evolution for oral powders and solutions (+ 8% of nDD/animal) and injectable forms (− 4% of nDD/animal). The consumption of four antimicrobial classes was significantly reduced (*p* < 0.05): lincosamides (− 51%), tetracyclines (− 32%), polymyxins (− 22%) and macrolides (− 19%).

**Fig. 1:**
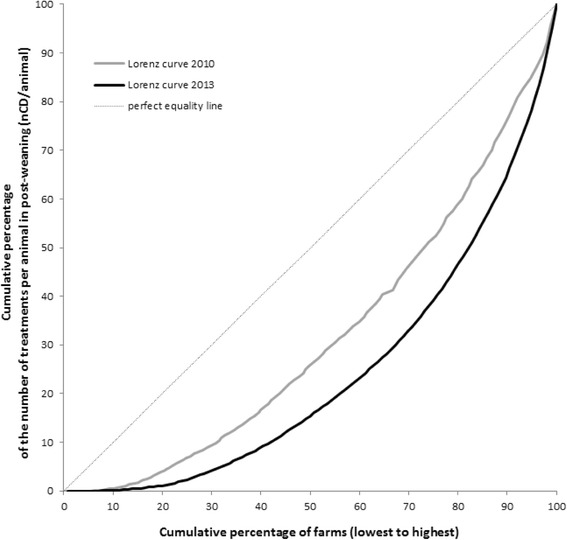
Distribution of postweaning uses (nDD/animal) in the 2010 and 2013 samples of farms (Lorenz curves)

As the Lorenz curves show (Fig. 1, Table 2), 50% of the treatment courses were performed by 25% of farms in 2010 and 18% in 2013, this reduction of the proportion of high-consuming farms was also observed for other production stages (Table 2).

### Evolution of usage in fatteners

Antimicrobial usage in fatteners decreased between 2010 and 2013 (− 29% of nDD/animal), without reaching statistical significance (*p* = 0.09). Again, the use of medicated feed has been significantly reduced for both indicators: nDD/animal (− 64%, *p* < 0.05) and proportion of farms using this mode of treatment (from 29 to 16% of the farms in the respective samples). There is a strong, although non-significant, increase in the usage of oral powders and solutions (+ 54% of nDD/animal).

Although four out of five antimicrobial classes displayed a numerical reduced usage, this reached statistical significance in a single family (i.e. penicillins, − 14%).

### Evolution of indications for treatment

There was a significant increase in the proportion of farms where sows were treated for locomotor disorders (Table 2) between 2010 and 2013. This indication remained however limited (3% of the 2013 nDD/animal), as compared to the dominant indication for antimicrobial treatment (urogenital disorders, 65%). The motivations for treatment were not significantly affected either, except for the locomotor disorders (+ 150% of nDD/animal between 2013 and 2010, *p* < 0.05).

In suckling piglets, the frequency of the dominant indications (respiratory and locomotor disorders) decreased (− 22% and - 17%, respectively of nDD/animal), without reaching statistical significance. However, the proportion of farms where such treatments took place remained stable (respectively 64% and 58% in 2013). Regarding post-weaning, a significant drop occurred in the proportion of uses for digestive disorders (− 29% of nDD/animal) that are the dominant indication (62% of the amounts used in 2013). The proportion of farms where these treatments were performed did not significantly change (88% of concerned farms in 2013). Conversely, even though the proportion of uses for locomotor diseases decreased (− 78% of nDD/animal), the proportion of treated farms increased significantly (from 29 to 51%). In finishers, the two dominant indications for treatment (digestive and respiratory disorders) did not present significant changes between 2010 and 2013.

## Discussion

### Representative sample of farms methodology

The strong positive response rate of the farmers to the request for participating in the samples, observed both in 2010 and 2013 (> 75%), while no counterpart was offered in our protocol, reflects a lasting strong motivation of farmers in supporting the study and tackling the issue of veterinary antimicrobial consumption. This also indirectly reflects the strong empowerment by the production organisations and the swine veterinarians in France on this subject. This is further substantiated by the initiative from swine industry representatives (both farmers and veterinarians) of a voluntary moratorium on 3rd and 4th generation cephalosporins in swine production. Announced in November 2010, this moratorium entered into effect by spring 2011, and was subject to wide national media coverage. On the other hand, the French ministry of Agriculture launched in June 2012 a plan to reduce by 25% veterinary antimicrobial consumption within 5 years, EcoAntibio 2017,^5^ and this might also have positively influenced the enrolment of farmers in the 2013 sample.

All the quantitative 2010 and 2013 data were harvested from reliable and comprehensive sources: acquired antimicrobial quantities were provided by the dispensing structure for each farm, from the list of providers that had been issued by each farmer, and yearly swine production was extracted from the farmers’ accounts.

The representativeness of each sample with the national swine herd population was verified against data from the two most recent agricultural censuses (2010 and 2013). Both samples being found representative, this provides robustness to the data on antimicrobial usage collected in both these instances. This allowed comparing the results obtained in each of the samples (evolution), and also with the national sales data, monitored by the French authorities.

### Total usage evolution between 2010 and 2013

In both years studied, the ALEA calculation of total usage of antimicrobials in the samples was found different from that estimated by the French authorities. This disparity is reduced for the 2013 data (15.6% difference) as compared to 2010 (27.6% difference). It is striking that in both years, the calculation indicates a lower swine exposure to antimicrobials than the national estimates do. In the case of the sales monitoring, the attribution to the swine species is indicated by the manufacturers, while the sample of farms methodology collects on-farm usage data. As such, the outcome of the latter is most probably closer to the actual antimicrobial swine exposure level at national level.

In fact, the single important difference between the samples’ and authorities’ ALEA values is for tetracyclines — suggesting that the sales of this antimicrobial class to the swine industry are overestimated by the pharmaceutical industry in their notification to the French Veterinary Medicines Agency, possibly because most corresponding VMPs are registered for a multi-species usage.

In any case, both sources concur in confirming a highly significant drop in antimicrobial consumption in the French swine industry over the three years considered. This is consistent with the recent comparison of three methods for stratifying antimicrobial sales data per animal species in Switzerland (equal distribution, biomass distribution and longitudinal study extrapolation), in reference to a detailed recording system of prescriptions [11], where all methods found comparable trends, with marginal differences regarding the swine species. Furthermore, a cross-sectional study, based on voluntary participation of swine veterinarians and farmers, was successfully performed in Germany for the year 2011, providing results in line with national antimicrobial sales data [12].

However, the 7% reduction in the value of the ALEA observed thanks to the sample methodology between 2010 and 2013 does not accurately reflect the extent of the changes in antimicrobial usage in suckling piglets, weaned pigs and finishing pigs, the dominant stages as far as antimicrobial consumption is concerned. It may largely be explained by the sensitivity of this indicator to the treatments ascribed to sows, since it processes a percentage of treated liveweight: a 300-kg sow will have a one-hundred-fold more important impact on the ALEA than the treatment of a 3-kg pig would. Since the 2013 sample recorded a (non-significant) increase in sows’ treatments, the drop in total exposure was – arithmetically – limited through this methodology. These considerations on the reduction of usage of antimicrobial VMPs in France are indirectly substantiated by the recently published study of the evolution of animal health expenses in swine farms [13]. After having plateaued between 2006 and 2011, treatment products expenditures (in €) dropped by 18% within two years (− 20% for in-feed supplementations and − 15% for parenteral products) while vaccine expenses increased by 10%. Although that study extends from 2002 to 2012 in farrow-to-finish French farms and that expenses are not explicitly related to amount used, it evidences that significant changes occurred between 2010 and 2012. In Denmark, an increase of vaccine usage has also been observed concurrently with a decrease in antimicrobial consumption that followed the announcement and introduction of the Yellow Card intervention [14], even though the use of vaccines does not explain per se a drop in antibiotic usage [15].

The decrease in total antimicrobial consumption in France reflects the positive attitude of swine farmers and practitioners toward reducing antimicrobial usage. It is temporally associated with wide and extensive national media coverage of the French Ministry of Agriculture’s announcements on the national plan on reduction of veterinary antimicrobial usage; such a temporal association has also been observed in Denmark [13] and the Netherlands [16].

For the moment, this reduction has mostly been achieved through a partial shift in the treatment mode, with a strong and highly significant reduction of in-feed supplementation for weaners (− 25% of nDD/animal between 2013 and 2010) and a lower – non-significant – increase of drinking-water soluble products used postweaning (+ 8% nDD/animal).

The units in which national reports on antimicrobial consumption are presenting yearly results have also long been left un-harmonised, hampering international comparisons [5, 17–20]. The recent proposal of harmonised DDDvet and DCDvet units in Europe [8] will resolve this issue for the calculation of the numerator of the concerned indicators. However, it can be observed that in Germany in 2011 [11] tetracyclines were quantitatively the most commonly used antimicrobials on swine farms, while in France they ranked second in 2013. In another cross-sectional study in four European member states, where data collection took place in 2012–2013, aminopenicillins were found to be the antimicrobial class the most commonly used in all countries [21]. However, indicators differ and the numbers of sampled farms were more limited than in the present French and the recent German [11] studies.

### Usage in sows

This study evidenced a trend of increasing antimicrobial usage in sows (+ 17% of nDD/animal) over the 3-year period. Although not of absolute evidence, the impact of transition to sow group-housing (Council Directive 2008/120/EC) seems to have been the main driver of this evolution. It is well known that the French swine industry was behind schedule to implement this European regulation, as were 5 other Member States [22]. This has resulted in extensive and widespread building works in farm facilities over 2012–2013, which might have disturbed farms’ management and routine – and stressed the animals. The observed increase in most treatment motivations is in favour of such a temporary unbalance of the farms’ global sanitary situation around 2013. Additionally, a cross-sectional study involving 108 farms in western France identified group-housing in large groups of sows as a significant risk factor associated with leg problems [23]. Also, in German study, where transition to group-housed sow breeding had been performed with more anticipation, the main indication for treatment in sows was found to be respiratory diseases; not leg disorders [11].

Taken together, these elements may explain the significant increase in treatments for locomotor disorders observed in 2013 in sows (+ 150%) on a wide array of farms (+ 35%).

Also, an increase in the incidence of clinical leptospirosis has been described in association with loose-housing of sows in Denmark [24]. This was reported as having resulted in a wide usage of leptospirosis vaccination in sow herds. In France, no commercial vaccine was available for prevention of leptospirosis in sows at the time of the study, but the 17% increase observed in 2013 for genito-urinary disorders and the parallel increase in the usage of tetracyclines in sows might at least partly reflect an increase in leptospirosis control strategies.

After this adaptation phase, follow-up studies should allow to evaluate whether antimicrobial consumption of sows remains at the 2013 level, or whether it follows the decreasing trend observed in the other production stages. A 2016 sample of farms has recently been constituted and is being subject to interviews at the time of submission of the present manuscript, in accordance with the herein detailed protocol.

### Usage in suckling piglets

Antimicrobial usage has been significantly reduced between 2010 and 2013, the strongest drop being for cephalosporins. None of the treatment indications frequency increased over that period, which might suggest that the change in antimicrobial usage was accompanied by changes either in piglet management/care or in sow preparation, or both.

The bottom-up approach chosen for the present study allows to discriminate exposure levels in sows and suckling piglets, which is not performed in other national monitoring programmes such as Denmark [25] or the Netherlands [26] hampering comparison of results.

Finally, one of the main objectives fixed by Inaporc at the start of the study was reached: the voluntary ban on 3rd and 4th generation cephalosporins that started in spring 2011 was widely implemented on French farm, in a measurable way.

### Usage in weaned pigs

Postweaning remains the most antimicrobial-exposed production stage, as in other European countries, whatever the methodology used for monitoring [11, 20, 26]. Between 2010 and 2013, this production stage benefited from the decreased use of in-feed antimicrobial supplementation (i.e. medicated feed, since top-feeding is not allowed in France), and although the proportion of farms using polymyxins remained high, there was a 22% drop in its usage (in nDD/animal) over the period. Most remarkably, all antimicrobial classes were found to have a decreased usage (statistically significant for 4 out of 6 of them) — with the notable exception of potentiated sulphonamides (+ 59%), whose increase did not reach statistical significance. This impact of the putative switch from in-feed to water administration of antimicrobials could be expected since the duration of each treatment course [27] (and corresponding number of daily dose) is more limited through water medication than direct in-feed manufacturing.

In the present case, although digestive disorders remained the dominant cause of postweaning treatments, their relative importance regarding antimicrobial consumption of this production stage dropped significantly. The usage of zinc oxide had not been authorised before 2016 in France, and a new vaccine against oedema disease had just reached the market in 2013. It can safely be inferred that treatment strategies of digestive disorders have profoundly changed during the study period, less relying on in-feed supplementation over extended durations, and more on short-term targeted treatments via drinking water.

### Usage in fatteners

As for weaners, usage of in-feed supplementation was strongly (and significantly) reduced among finishers between 2010 and 2013. The main difference was that only a single class of antimicrobials was significantly affected (i.e. penicillins), whereas tetracyclines, which are the main class of antimicrobials used at this production stage, were not.

The dominant indication for treatment were the respiratory diseases in finishers, both in 2010 and 2013, which is consistent with the results of the German survey performed with the 2011 data [12], but is different from the outcome of a retrospective Danish study where gastro-intestinal dominated the indications for treatment for the 2002–2008 period [28]. However the frequency of respiratory diseases as an indication for treatment was on an increasing trend (+ 81% per finishing pig produced) while that of gastro-intestinal diseases was decreasing, which clearly places this indication as an emerging clinical entity. Also, large variation in antimicrobial usage patterns have been described between European countries, as analysed for the 2008–2013 period between Denmark and Switzerland [29].

### Limitations of the study

Although all treatments are compulsorily recorded by the farmer in the farm logs, as required by EU and national^6^ regulations, the description of antimicrobial usage may be subject to memory bias, since interviews were conducted on the year following actual usage (end of 2011 and early 2012 for the 2010 sample survey; 2014 for the 2013 one). This bias is however limited since it can affect the attribution of the treatment to one of the other of the production stages, but not the quantities used on the farm. Also, this bias is almost excluded for in-feed supplementation, since all production stages are clearly identified through the nature of the feed delivered to the farms. The motivation for treatments was more prone to such a recall bias; in a limited extent however: in 6% farms, the motive of suckling piglets’ treatment could not be defined by the farmers, and this proportion was at most 12% for the post-weaning stage, in 2010. For 2013, these proportions were nil regarding sows and suckling piglets, and 4 and 3% in post-weaning and finishing stages, respectively.

## Conclusion

Important and significant drops of antimicrobial usage were evidenced between 2010 and 2013, which comply with the national reduction target set up in 2012 of − 25% by 2017. These results reflect a strong sensitisation of swine production professionals to the antimicrobial reduction objectives, and are the result of pre-2012 voluntary measures, such as the moratorium on the use of 3rd and 4th generation cephalosporins. This sample methodology is currently renewed, in order to validate that the national targets have been met, and whether the reduction of post-weaning usages (partial substitution of in-feed medication by more occasional – and judicious – drinking water treatment) has remained in place, or even exceeded expectations.

## Abbreviations

ALEA: Animal Level of Exposure to Antimicrobials
ANMV: Agence Nationale du Médicament Vétérinaire (French Agency for Veterinary Medicinal Products)
ANSES: Agence nationale de sécurité sanitaire de l’alimentation, de l’environnement et du travail (French Agency for Food, Environmental and Occupational Health & Safety)
BDPORC: Base de Données professionnelles PORCine (French swine database of identification)
CD: Course dose
DD: Daily dose
ESVAC: European Surveillance of Veterinary Antimicrobial Consumption
IFIP: Institut technique de recherche et de développement de la filière porcine (swine industry’s French technical institute)
INAPORC: Interprofession nationale porcine (French Interprofessional Pork Council)
SPC: Summary of Product’s Characteristics
VMP: Veterinary Medicinal Product

## Footnotes

1 [in French]. Chouët S, Delsart M, Deville N, Dréau D, Lannou J, Lemistre A, Liber M, Marchand D, Normand V, Sevin JL, Sialleli JN. Consensus sur l’utilisation des céphalosporines de 3^e^ et 4^e^ générations en pathologie porcine. *Bulletin des GTV*, 2012 (64):55–56.

2 Study by Chauvin, published by the French Food Safety Agency, Anses, in 2010, available (in French) at https://www.anses.fr/fr/system/files/LABO-Ra-AntibioEtudPorcin.pdf

3 BDPORC has a public informative website (in French) at http://www.bdporc.com/alertes/accueilAbonne.do

4 Most swine French farmers use a common performance data programme, developed, distributed and operated by IFIP. See at http://www.ifip.asso.fr/fr/resultats-economiques-gttt-graphique.html (in French)

5 For more information on the Plan EcoAntibio 2017 (in English), see: http://agriculture.gouv.fr/sites/minagri/files/documents/pdf/130208PlanABR-GB-2012-BD_cle8786a1.pdf (32 pages document). At the time of submitting this manuscript, this target has been reached.

6 See French regulation: https://www.legifrance.gouv.fr/affichTexte.do?cidTexte=JORFTEXT000000583065 (in French).
